# In-ear electroencephalography: a systematic review of technology, applications, challenges, and future directions

**DOI:** 10.3389/fneur.2026.1914159

**Published:** 2026-10-27

**Authors:** Chirag Haria, Benjamin H. L. Harris, Lucy Mortimer, Katarzyna Platek-Johnson, Simon Freilich, Louis J. Koizia, Michael B. Fertleman

**Affiliations:** 1 Cutrale Perioperative and Ageing Group, Department of Bioengineering, Imperial College London, London, United Kingdom; 2 St. Catherine’s College, University of Oxford, Oxford, United Kingdom; 3 Department of Oncology, University of Oxford, Oxford, United Kingdom; 4 Division of Medicine, Institute for Liver and Digestive Health, University College London, London, United Kingdom; 5 Department of Psychosocial Rehabilitation, Medical University of Lodz, Lodz, Poland; 6 Collaborating Centre for Values-based Practice, St. Catherine’s College, University of Oxford, Oxford, United Kingdom; 7 Luton & Dunstable University Hospital, Luton, United Kingdom

**Keywords:** brain monitoring, ear-EEG, in-ear EEG, systematic review, wearable EEG

## Abstract

Electroencephalography (EEG) is widely used to monitor brain activity in both clinical and research settings. However, conventional scalp EEG systems require multiple electrodes, specialised equipment, and trained personnel, which limits their practicality for long-term monitoring in everyday environments. In-ear EEG has emerged as a promising wearable alternative that enables discreet and portable neural monitoring. This systematic review examines the current state of research on in-ear EEG technologies. Twenty-four studies met the inclusion criteria and were analysed thematically. The literature highlights rapid progress in device miniaturisation and wearable design, with reported applications including sleep monitoring, seizure detection, cognitive workload assessment, fatigue monitoring, and emotion recognition. Despite these advances, challenges remain related to signal quality, spatial coverage, and motion artefacts. Further research is required to validate in-ear EEG for broader clinical and real-world applications.

## Introduction

The electroencephalogram (EEG) is one of the most widely used techniques for recording the electrical activity of the brain and plays a crucial role in both clinical practice and neuroscience research (1). By placing electrodes on the scalp, EEG enables the non-invasive monitoring of neural activity and has become an essential tool for diagnosing and studying neurological disorders such as epilepsy, sleep disorders, and brain injuries. Conventional scalp EEG systems are the most established approach and have been extensively validated for clinical monitoring and research purposes (1). Despite their effectiveness, these systems typically require multiple electrodes, specialised equipment, and trained personnel for installation, which limits their practicality for long-term monitoring outside controlled clinical environments.

In recent years, rapid advances in wearable sensing technologies and miniaturised electronics have led to the development of portable physiological monitoring devices. These innovations have facilitated continuous monitoring of biological signals in everyday environments, contributing to the growing field of digital health. Among these technologies, wearable devices positioned in or around the ear, commonly referred to as earables, have attracted increasing attention as platforms for physiological sensing (2). Due to their unique location on the head, ear-based devices can be used to measure a range of physiological parameters including head and eye movements, cardiovascular activity, respiration, and other body signals (3, 4). Importantly, ear-mounted devices are typically discreet and comfortable, often resembling commonly used consumer electronics such as earbuds or hearing aids.

The anatomical proximity of the ear to the brain has also made it a promising site for monitoring neural activity. This has led to the development of in-ear electroencephalography (in-ear EEG) systems, in which electrodes are placed within or around the external auditory canal to capture brain signals (5). In the early 2010s, a study demonstrated that EEG signals could be successfully recorded using electrodes embedded within custom ear moulds positioned in the external auditory canal (6). Since this initial demonstration, research into ear-EEG technologies has expanded rapidly, exploring different device architectures, electrode materials, and recording configurations (3, 7). In-ear EEG systems typically employ a small number of electrodes integrated into earbud-like devices, enabling more convenient and discreet recording compared with conventional scalp-based EEG systems. Over the past decade, the number of studies investigating ear-EEG systems has increased substantially, reflecting growing interest in wearable neurotechnology for continuous brain monitoring.

One of the key advantages of in-ear EEG technology is its portability and potential for long-term monitoring. Unlike traditional EEG systems, which require extensive setup procedures and conductive gels, in-ear devices can often be inserted similarly to standard earphones and used without specialist assistance. The stable positioning of the ear canal may also help maintain consistent electrode contact and reduce certain types of motion artefacts (8). As a result, in-ear EEG systems have been investigated for a wide range of applications, including emotion recognition, stress monitoring, objective hearing assessment, and drowsiness detection (9–12). In medical contexts, they have shown promise for long-term monitoring of sleep stages and epileptic seizures outside hospital environments (13–15), as well as for monitoring neurological conditions in Alzheimer’s disease and Lewy body dementia (16, 17). The development of wearable neurotechnologies also aligns with broader efforts to integrate digital health tools into clinical care pathways and improve patient-centred care and innovation (18, 19).

Despite these advantages, several challenges remain in the development and implementation of in-ear EEG systems. Compared with conventional scalp EEG, ear-based recordings generally rely on fewer electrodes and provide limited spatial coverage of the brain. This can lead to lower signal amplitudes and reduced spatial resolution (20). Additionally, the quality of recordings can be influenced by factors such as electrode-skin impedance, motion artefacts caused by facial or head movement, and variability in ear canal anatomy between individuals (21, 22). These technical constraints continue to motivate ongoing research into improved electrode materials, device design, and advanced signal processing techniques aimed at enhancing signal reliability and usability.

Given the rapid expansion of research in this area, a comprehensive synthesis of existing studies is necessary to better understand the capabilities and limitations of in-ear EEG technology. This systematic review therefore aims to examine the current state of research on in-ear EEG systems. In particular, this review focuses on four key aspects: (1) technological development of in-ear EEG devices, (2) signal quality and comparison with conventional scalp EEG, (3) current clinical and cognitive applications, and (4) the limitations and future directions of this emerging technology. By summarising the evidence across existing studies, this review seeks to provide a clearer understanding of the potential role of in-ear EEG in future wearable neurotechnology and real-world brain monitoring.

## Methods

### Search strategy

This systematic review was conducted in accordance with the general principles of the Preferred Reporting Items for Systematic Reviews and Meta-Analyses (PRISMA) guidelines. A systematic literature search was conducted to identify peer-reviewed studies related to in-ear EEG. The databases PubMed and Google Scholar were used to retrieve relevant publications investigating the development, evaluation, or application of in-ear EEG systems. The literature search was conducted in January 2026. The search strategy used combinations of keywords including “in-ear EEG,” “ear-EEG,” “wearable EEG,” “EEG electrodes,” “brain–computer interface,” and “EEG signal quality.” Boolean operators AND and OR were used to combine terms during the search process. Example search combinations included “in-ear EEG OR ear-EEG” AND “wearable EEG” and “in-ear EEG” AND “brain–computer interface.” The term “brain–computer interface” was included to maximise retrieval of studies describing in-ear EEG technologies within the broader wearable neurotechnology literature, rather than to specifically identify brain–computer interface applications. All retrieved studies were subsequently assessed against the predefined eligibility criteria, and studies without an in-ear EEG component were excluded during screening. Only articles published from 2018 onwards were included in order to capture recent developments in wearable neurotechnology and in-ear EEG device design as the field remained a relatively small niche in its early years. Additionally, only articles published in English were considered for inclusion in the review.

### Selection criteria

Studies were selected according to predefined inclusion and exclusion criteria.

Articles were included if they:

- Investigated the development, testing, or implementation of in-ear EEG systems
- Evaluated signal acquisition methods or signal quality in ear-based EEG recordings
- Compared in-ear EEG systems with conventional scalp EEG systems
- Reported applications of in-ear EEG, including clinical, cognitive, or monitoring contexts
- Reported either experimental studies, clinical investigations, or technological validation studies.

Articles were excluded if they:

- Focused primarily on other EEG modalities without relevance to in-ear EEG technology
- Did not involve experimental or technological evaluation of ear-based EEG systems
- Were non-peer-reviewed publications or lacked sufficient methodological detail

### Study selection and data extraction

The screening process consisted of two stages. First, titles and abstracts were reviewed to determine whether the articles were relevant to the topic of in-ear EEG technology. Studies that appeared relevant were then assessed through full-text evaluation to confirm whether they met the predefined inclusion criteria. Where uncertainty existed regarding eligibility, studies were reviewed against the predefined inclusion and exclusion criteria before a final decision was made. Following this process, articles were identified and included in the final review. Each selected study was analysed in detail in order to identify key findings and recurring themes.

Data from each included study were extracted using a standardised data extraction framework developed for this review. Information extracted from the selected studies included:

- Technological design and development of in-ear EEG devices
- Signal acquisition methods and signal quality characteristics
- Comparisons between in-ear EEG and traditional scalp EEG systems
- Clinical, cognitive, or experimental applications of in-ear EEG
- Participant demographics (where reported)
- Reported limitations and future research directions

The extracted data were analysed using a thematic approach, allowing key research themes across the literature to be identified. These themes included technological development, signal quality and performance, emerging applications of in-ear EEG, and current technical challenges. The methodological strength of the included studies was also assessed using a review specific framework adapted from the Oxford Centre for Evidence-Based Medicine (CEBM) Levels of Evidence, providing a structured assessment according to study design.

## Results

A total of 24 studies met the eligibility criteria and were included in the final analysis. The overall study selection process is illustrated in the PRISMA flow diagram (Figure 1).

**Figure 1:**
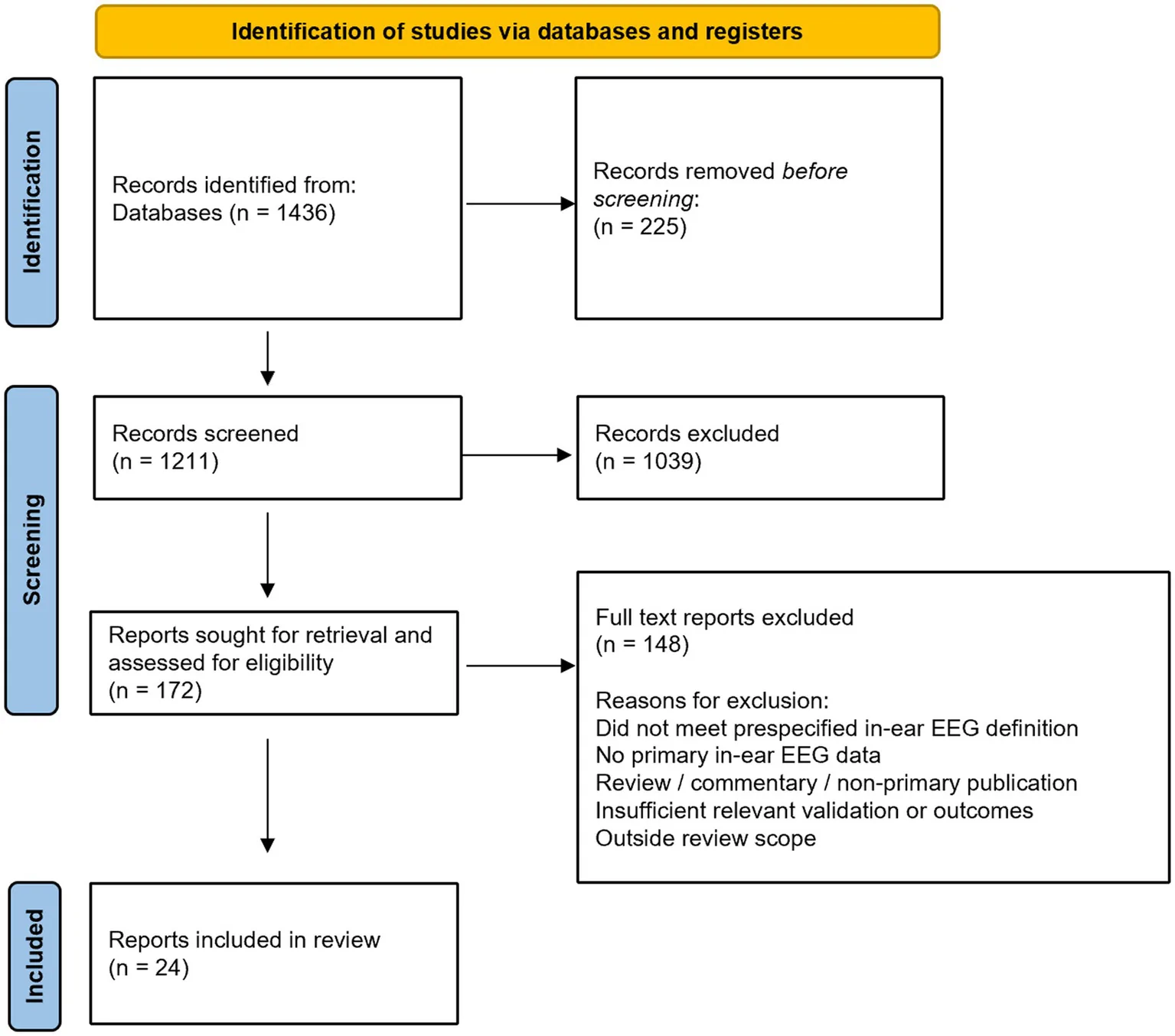
PRISMA study selection flow diagram. Illustration of the study identification, screening, eligibility assessment, and inclusion process used in this systematic review. The diagram outlines the number of records identified through database searches, records screened, reports assessed for eligibility, and the final studies included in the review following the PRISMA framework.

### Technological development of in-ear EEG

The development of in-ear EEG has been driven by the need for compact, comfortable, and portable systems capable of monitoring brain activity outside conventional clinical environments. Early work by Looney et al. established the feasibility of recording EEG signals from electrodes positioned within the ear canal, marking an important step in the development of wearable EEG systems (6). Subsequent research has focused on improving device miniaturisation, comfort, and signal stability.

Current in-ear EEG systems are typically designed as small, lightweight, and discreet devices, often integrated into earbud-like earpieces or modified earphones, as illustrated by the exemplar device shown in Figure 2. Compared with conventional scalp EEG, these devices generally use fewer electrodes, commonly one to three channels, and primarily capture signals from regions close to the temporal lobe. Their portability and ease of use make them attractive for prolonged monitoring in naturalistic settings (23).

**Figure 2:**
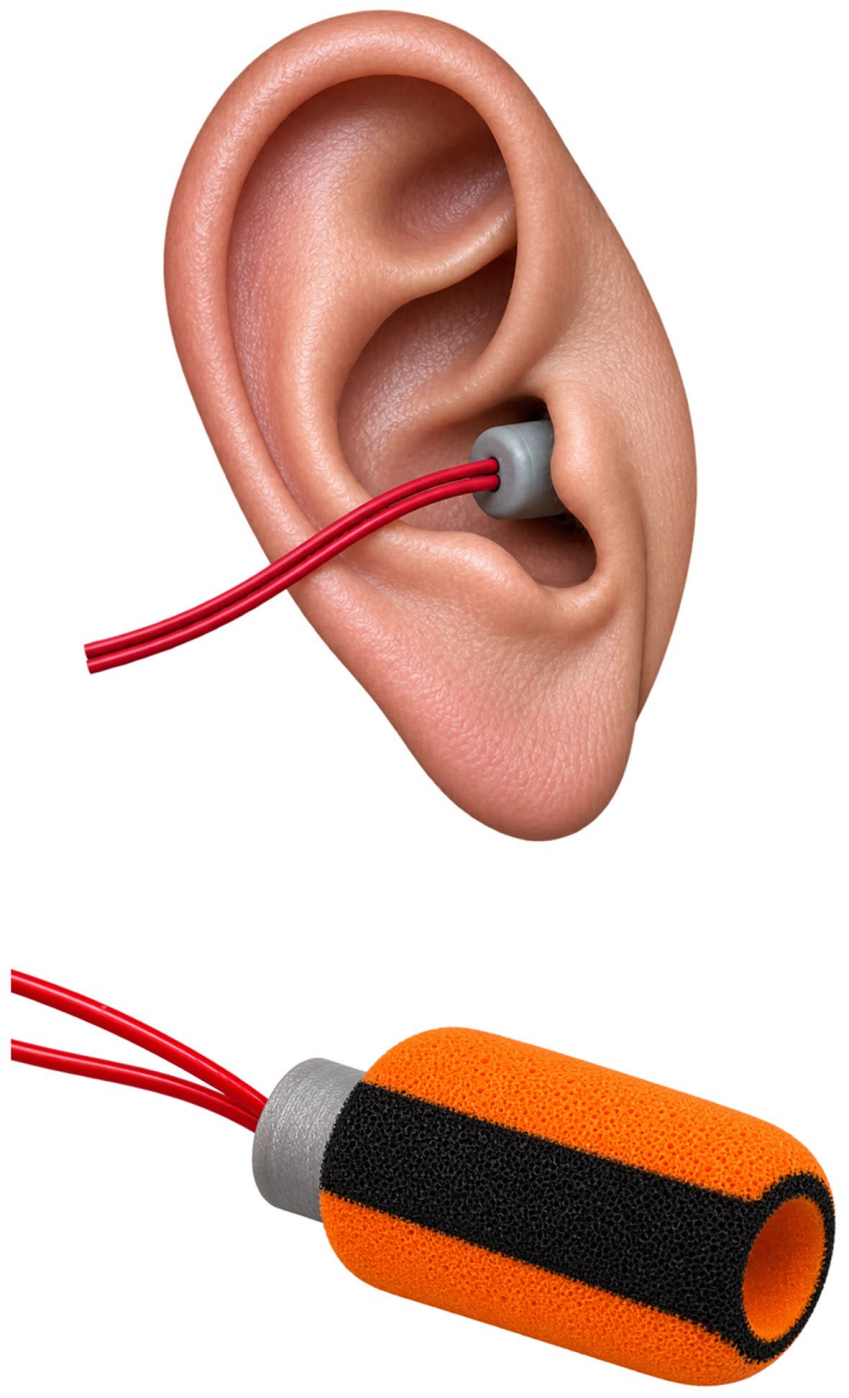
Illustrations of an in-ear EEG device. The upper image shows the device worn within the ear, illustrating its low-profile design, anatomical fit within the concha and external auditory canal. The lower image shows the assembled device, composed of conductive elastomer-coated foam electrodes with connecting leads.

Design innovation has also centred on improving fit and wearability. Custom-moulded earpieces have been developed to improve electrode-skin contact and reduce discomfort caused by variation in ear canal anatomy (24). Other studies have explored more ergonomic earbud-like designs intended to improve wearability and signal stability during daily activities (23, 25, 26). Despite these advances, signal acquisition remains a major technical challenge. Because in-ear systems use fewer electrodes and record from a restricted anatomical region, their signals are generally weaker than those obtained with conventional scalp EEG, resulting in lower signal-to-noise ratios (23, 27). In addition, variation in ear shape between users can affect fit, electrode contact, and reproducibility of recordings. Motion artefacts caused by head and facial movement also remain an important obstacle, although newer systems increasingly incorporate signal processing approaches to reduce these effects (28).

### Applications of in-ear EEG

Recent reviews suggest that in-ear EEG has potential across a broad range of clinical and non-clinical applications including medical monitoring, cognitive assessment, emotion recognition, and neurofeedback (5, 29). In healthcare, one of the most promising uses is long-term monitoring in settings where conventional scalp EEG is impractical. Sleep monitoring has been studied extensively, with several reports suggesting that in-ear EEG can provide useful information on sleep stages and may correlate well with conventional scalp EEG or polysomnography (15, 30). For example, Moumane et al. reported that although in-ear EEG signals had lower amplitudes than scalp EEG, they were still able to provide meaningful information about sleep related characteristics (27), supporting earlier work demonstrating strong correlations between ear-EEG and scalp EEG recordings during sleep (15). This supports the potential of in-ear EEG as a less intrusive tool for home-based and long-term sleep monitoring. Other work has also suggested that ear-EEG may be suitable for long-term home-based monitoring outside traditional laboratory environments (31).

Epilepsy monitoring is another important area of application. Zibrandtsen et al. (13, 32) reported that in-ear EEG detected focal-onset seizures and generalised tonic-clonic seizures with promising sensitivity and a low false detection rate, suggesting potential for ambulatory seizure monitoring. Other work has explored the use of in-ear EEG in identifying abnormal brain activity in neurological disorders, including the detection of subclinical epileptiform discharges and abnormal neural activity (16, 17). These findings further support the potential role of in-ear EEG in long-term remote neurological monitoring.

Beyond clinical monitoring, in-ear EEG has shown value in cognitive and behavioural applications. Several studies have investigated its use in cognitive workload and attention state estimation (20, 33– 35). Cognitive workload monitoring is particularly relevant in high-stakes environments such as aviation, driving, and military operations where performance may be affected by mental fatigue. Tremmel et al. (33) found that in-ear EEG could distinguish between lower and higher workload states, although with lower accuracy than conventional scalp EEG. Ear-EEG has also been explored for monitoring mental load, fatigue detection during driving and other attention-demanding activities, where changes in EEG alpha-band activity may indicate reduced alertness (26, 36).

Emotion recognition represents another emerging use. Athavipach et al. (9) reported that in-ear EEG could classify emotional states based on valence and arousal with performance comparable to scalp EEG recordings from temporal sites. Additional studies suggest that in-ear EEG may support applications in mental wellness, mood monitoring, and human-computer interaction (9, 10, 34, 35). Similarly, work in brain-computer interfaces (BCIs) indicates that ear-based EEG may provide a practical non-invasive platform for attention monitoring and user-state detection (35). Together, these findings indicate that in-ear EEG may be particularly useful where discreet, real-time, and continuous monitoring is required.

### Signal quality and comparison with conventional scalp EEG

A consistent finding across the literature is that in-ear EEG offers important practical advantages over conventional scalp EEG, particularly in terms of portability, comfort, and suitability for prolonged use. Unlike scalp EEG systems, which require multiple scalp electrodes, conductive gel, and trained personnel, in-ear devices can often be self-inserted and worn for longer periods in everyday settings. This makes them especially attractive for ambulatory monitoring and wearable neurotechnology applications.

However, these practical advantages are accompanied by technical trade-offs. The most frequently reported limitation is reduced signal quality relative to scalp EEG. Because in-ear devices typically use a small number of electrodes positioned within the ear canal, they provide narrower spatial coverage and generally lower signal amplitudes than conventional scalp recordings. Moumane et al. reported lower signal-to-noise ratios for in-ear EEG than for scalp EEG, particularly during more dynamic or demanding tasks (27). Similarly, Crétot-Richert et al. (20) highlighted lower signal quality and reduced sensitivity for certain signal components, particularly in the time domain.

Spatial resolution is also limited. Conventional scalp EEG provides broader coverage across cortical regions, whereas in-ear EEG primarily captures activity from areas near the temporal lobe. This restricted coverage may reduce performance in tasks involving distributed brain networks or more complex cognitive processes (33). Nevertheless, studies suggest that in-ear EEG remains sufficiently informative for targeted applications such as workload estimation, sleep analysis, fatigue monitoring, and emotion detection (29). Some recent work has attempted to improve this limitation by increasing electrode density around the ear. For example, Meiser et al. (37) reported that higher-density ear-EEG systems improved spatial sampling and enhanced performance in measuring auditory evoked and event-related potentials.

Motion artefacts are another major concern. In contrast to scalp EEG systems secured with caps or headbands, in-ear EEG depends on stable contact between the earpiece and the ear canal. Head movement, facial muscle activity, jaw motion, and speaking can all disturb electrode positioning and degrade signal quality. These issues appear to be particularly relevant during dynamic cognitive tasks, whereas signal quality is generally better under more passive conditions such as rest or sleep (27, 33).

### Current limitations and future directions

Across the reviewed literature, the main limitations of in-ear EEG relate to signal quality, motion artefacts, anatomical variability, and usability. Reduced signal-to-noise ratio remains a central challenge, especially during real-world movement. The small number of electrodes and limited coverage of the ear region constrain spatial resolution and may reduce the ability of in-ear EEG to capture more distributed brain activity. In addition, variability in ear canal shape and size between individuals affects fit, electrode contact, and consistency of measurement. Although custom-fitted devices can improve performance, they also increase manufacturing complexity and may limit scalability.

Comfort and usability are also important considerations for long-term monitoring. Although in-ear EEG is generally more discreet and portable than scalp EEG, prolonged wear may still cause discomfort, pressure, or irritation in some users (27, 29). Battery life and the need for lightweight but durable hardware also remain practical concerns for prolonged use in daily life.

Future development is likely to focus on improving signal fidelity, comfort, and real-world robustness. Advances in electrode materials and design may help improve electrode contact and reduce impedance. Greater use of multi-electrode ear arrays may also improve spatial resolution while preserving wearability. In parallel, advances in adaptive filtering, independent component analysis, wavelet-based denoising, and machine learning may improve artefact rejection and enable more reliable real-time analysis. Additional integration with wireless systems and multimodal wearable sensors may further support the transition of in-ear EEG from a promising experimental technology to a practical tool for continuous brain monitoring in both clinical and non-clinical settings. Future developments are also expected to focus on the creation of fully wireless and miniaturised in-ear EEG systems capable of continuous monitoring in everyday environments (25, 26).

## Discussion

The findings of this review highlight both the growing interest in in-ear EEG technologies and the ongoing challenges associated with their clinical implementation. Overall, the reviewed literature suggests that in-ear EEG represents a promising direction in wearable neurotechnology, with the potential to enable continuous and unobtrusive monitoring of brain activity outside traditional clinical and laboratory environments.

A key advantage identified across studies is the portability and usability of in-ear EEG devices. Compared with conventional scalp EEG systems, which require multiple electrodes, conductive gels, and specialised installation, in-ear devices are compact, lightweight, and easier to deploy. These characteristics make them particularly attractive for long-term monitoring in ambulatory and real-world settings. The discreet design of ear-based devices, which often resemble common earbuds or hearing aids, may also improve user acceptability during prolonged use.

Across the literature, clinical monitoring appears to represent the most mature application area. Sleep analysis and seizure detection have been among the most frequently studied uses of in-ear EEG, with several studies demonstrating that ear-based recordings can capture neural signals relevant to sleep staging and neurological monitoring. These findings align with broader trends toward remote and home-based healthcare monitoring (15, 31). In-ear EEG may also have particular value for longitudinal monitoring in generalised epilepsy, including patients with infrequent or predominantly nocturnal seizures, where capturing events during conventional inpatient monitoring may be challenging. This may be particularly relevant for individuals at risk of sudden unexpected death in epilepsy (SUDEP). In-ear EEG may also support outpatient antiseizure medication adjustment by providing objective longitudinal seizure quantification alongside patient- or caregiver-reported seizure counts.

Beyond ambulatory monitoring, rapidly deployable in-ear EEG may also have potential in emergency and critical-care settings, where timely access to EEG can be important. Such systems could provide rapid screening for highly epileptiform patterns, including generalised periodic discharges and electrographic status epilepticus, helping to identify patients requiring escalation to conventional EEG monitoring. In addition, in-ear EEG may offer particular advantages in paediatric populations, where the small and unobtrusive form factor could improve the feasibility and tolerability of prolonged ambulatory or home EEG monitoring compared with conventional systems.

However, in-ear EEG is unlikely to replace conventional scalp EEG for comprehensive neurophysiological assessment because of its limited spatial coverage. Instead, it is better viewed as a complementary technology for applications where portability, continuous monitoring, and patient acceptability are prioritised over high spatial resolution, such as ambulatory monitoring, remote patient management, and home-based neurological assessment. In addition to clinical applications, several studies have explored the use of in-ear EEG in cognitive and behavioural monitoring, including cognitive workload assessment, fatigue detection, and emotion recognition. Such applications may support emerging fields such as neuroadaptive technologies, human–computer interaction, and digital health. The combination of in-ear EEG with artificial intelligence (AI) may further expand these applications by enabling automated interpretation of complex EEG signals, continuous personalised monitoring, and real-time detection of clinically relevant or behaviourally significant events.

Future research may also explore integrating wearable neurotechnology with genomic and multi-omics datasets, an approach increasingly used in other areas of medicine to better understand complex disease risk and support more personalised monitoring strategies (38–40). In addition, the development of standardised protocols and standard operating procedures, as implemented in other areas of clinical and translational research (41), may help improve reproducibility, data quality, and facilitate the wider clinical adoption of in-ear EEG technologies.

Despite these promising developments, several technical challenges remain. Compared with conventional scalp EEG, in-ear EEG generally provides lower signal amplitudes and reduced spatial coverage due to the limited number and placement of electrodes within the ear canal (42, 43). This restricted spatial sampling may be particularly relevant in extra-temporal or multifocal epilepsy, or in patients with mixed focal and generalised seizure types, where seizures arising from frontal, occipital, or other regions may be less readily detected. In addition, current in-ear EEG systems generally lack the synchronised video available with conventional video-EEG, which may limit electroclinical correlation and the distinction between epileptic, nonepileptic, and physiological events. Motion artefacts resulting from jaw movement, speech, or head motion may also affect signal stability. Anatomical variation in ear canal shape between individuals may influence electrode positioning and recording consistency, although custom-moulded earpieces may partially address this limitation. Electrode–skin impedance remains another important technical challenge, particularly during prolonged recordings. Early studies have demonstrated that physiological factors, including the presence of cerumen, can substantially increase dry-contact impedance and adversely affect signal quality (21). Although advances in electrode materials and signal-processing techniques have improved signal quality, comparatively little research has focused on optimising electrode preparation or systematically evaluating methods to reduce impedance. Future studies should prioritise the evaluation of practical impedance optimisation strategies to improve signal quality, recording reliability, and overall device performance. Furthermore, few studies have examined long-term user comfort, adherence, and device durability, despite these factors being essential for successful clinical implementation.

A limitation of the current evidence base is the limited representativeness of study populations. Most studies recruited small cohorts of young, healthy adults, often with gender imbalance, while relatively few included older adults or patients with neurological disease (Table 1). In addition, several studies did not report participants’ age or gender, limiting assessment of the generalisability and potential risk of selection bias. Although these populations are appropriate for early device development and technical validation, they may limit the generalisability of findings to the clinical populations most likely to benefit from continuous neurological monitoring. Future studies should therefore prioritise larger, more diverse and better-characterised cohorts to establish the clinical utility and external validity of in-ear EEG technologies.

**Table 1:** Summary of all studies identified and included in this review.

Author (year)	Study type	Participants	Primary application	Key findings	Technological contribution	Evidence level
Christensen et al. (2018) (11)	Experimental study	Healthy adults with normal hearing (*n* = 15) 11 male/4 female Mean age 30.3 years old; range 23–44 years old	Objective hearing threshold estimation	Hearing thresholds could be estimated with electrodes placed in one ear, although signal quality was somewhat lower than with scalp EEG.	Ear-EEG-based objective hearing assessment with potential for hearing-aid integration.	Level 3
Hong et al. (2018) (12)	Experimental machine learning study	Healthy adults (*n* = 16) 12 male/4 female Age range 25–32 years old	Drowsiness detection	Ear-EEG in combination with other modalities detected drowsiness during simulated driving with very high accuracy. Ear-EEG also performed well on its own.	Multimodal in-ear sensing system for drowsiness recognition.	Level 4
Zibrandtsen et al. (2018) (32)	Proof of concept study	Epilepsy patients (*n* = 21 total; ear-EEG cohort *n* = 15, scalp-EEG comparison cohort *n* = 6) Gender distribution not reported Age range not reported	Generalised tonic–clonic seizure detection	Ear-EEG derived signals detected all 3 recorded GTCS, with 100% sensitivity and no false positives supporting nocturnal seizure monitoring.	Proof of concept of ear-EEG for automated nocturnal generalised tonic–clonic seizure detection.	Level 4
Athavipach et al. (2019) (9)	Device development study	Healthy adults (*n* = 13) 12 male/1 female Mean age 24 years old; range 20–30 years old	Emotion monitoring	A low-cost, generic in-ear EEG prototype recorded neural signals similar to those from temporal scalp sites and showed moderate accuracy in classifying emotions. This supports its potential use for monitoring emotional states.	Low-cost generic in-ear EEG prototype for affective monitoring.	Level 4
Kappel et al. (2019) (42)	Computational modelling with experimental validation	Healthy adults (*n* = 4) 4 male/0 female Mean age 37 years old	Computational modelling and source mapping	Developed personalised computer models of the ear that can reasonably predict ear-EEG topographies. This model can help design and develop electrode configurations for wearable EEG systems.	Individualised forward modelling to optimise ear-EEG electrode configurations.	Level 4
Merrill et al. (2019) (24)	Experimental study	Healthy adults (*n* = 7) 7 male/0 female Age range not reported	Brain-based biometric authentication	This study demonstrated how ear-EEG can be used for security outside of clinical settings. Custom-fit in-ear EEG made it possible to use three-factor “passthought” authentication with high accuracy and very low false acceptance and rejection rates.	Custom-fit in-ear EEG for biometric authentication.	Level 4
Jeong and Jeong (2020) (35)	Experimental machine learning study	Healthy adults (*n* = 6) 4 male/2 female Mean age 28.17 years old; age range 25–30 years old	Attention-state classification	A custom in-ear EEG used with an echo state network could distinguish between attentive and resting states. This finding supports the use of portable devices for monitoring attention.	Machine-learning classification of attention using in-ear EEG.	Level 4
Kaveh et al. (2020) (25)	Device development study	Healthy adults (*n* = 3) Gender distribution not reported Age range not reported	Development of a wireless user-generic in-ear EEG system	Demonstrated use of a wireless ear-EEG device that recorded blinks, alpha rhythm and auditory steady-state response, although with lower signal-to-noise ratios compared to scalp EEG.	Wireless in-ear EEG acquisition system.	Level 4
Lee et al. (2020) (10)	Experimental machine learning study	Healthy adults (*n* = 10) Gender distribution not reported Mean age 29 years old	Stress monitoring	A headset combining in-ear EEG and ECG could distinguish between relaxed and stressed states with moderate accuracy.	Multimodal headset with dry in-ear EEG electrodes for stress monitoring.	Level 4
Nakamura et al. (2020) (15)	Validation study	Healthy adults (*n* = 22) Gender distribution not reported Mean age 23.8 years old	Sleep monitoring	Standardised in-ear EEG makes it possible to monitor sleep overnight and automatically predict sleep stages, with substantial agreement with polysomnography. This method could offer a less intrusive way to assess sleep in community settings.	Standardised ear-EEG platform for unattended home sleep monitoring.	Level 3
Sintotskiy and Hinrichs (2020) (31)	Device development study	Technical testing on 1 volunteer Gender distribution not reported Age range not reported	Home monitoring	Created a portable in-ear EEG device that connects to a smartphone which is affordable, uses little power, and provides a signal quality similar to standard EEG systems.	Smartphone-connected portable ear-EEG system for home use.	Level 4
Paul et al. (2021) (34)	Experimental machine learning study	Healthy adults (*n* = 3) 3 male Age range 23–31 years old	Attention-state classification	The authors used binaural in-ear EEG and a spatial separation technique to identify when people were attentive or resting, reaching moderate accuracy of about 70 to 75 percent. Although this is less accurate than scalp EEG, it suggests potential for portable cognitive monitoring.	Feature-based machine-learning approach for attention decoding from ear-EEG.	Level 4
Henao et al. (2022) (30)	Experimental study	Healthy adults (*n* = 24) 12 male/12 female Mean age 21.8 years old	Sleep monitoring	This study found that in-ear EEG can pick up slow oscillations during sleep. The results suggest that ear-EEG might be a more comfortable choice for sleep intervention systems.	Ear-EEG-guided closed-loop auditory stimulation during sleep.	Level 3
Mandekar et al. (2022) (23)	Device development study	Healthy adults (*n* = 10) 5 male/5 female Mean age 28 years old	Signal quality evaluation against forehead EEG	In-ear dry electrodes showed a strong alpha-band correlation with forehead EEG. This finding suggests that recordings from the ear canal could be a practical and portable option for some applications.	Validation of dry in-ear electrodes against forehead EEG.	Level 4
Occhipinti et al. (2022) (28)	Signal-processing methods study	Adults (*n* = 12) 8 male/4 female Age range 23–43 years old	Artefact removal for continuous monitoring	Suggested some signal processing techniques to remove motion and non-neural artefacts from ear-EEG recordings, although the authors considered the results inconclusive.	Signal-processing methods for motion artefact reduction during long-term ear-EEG monitoring.	Level 4
Crétot-Richert et al. (2023) (20)	Experimental study	Healthy adults (*n* = 15) 6 male/9 female Mean age 27.1 years old	Attention and cognitive workload monitoring	EEG signals collected in and around the ear can reliably detect differences in mental focus and cognitive workload. These results are similar to what is found with traditional scalp EEG.	Ear-centred EEG for cognitive workload assessment.	Level 3
Musaeus et al. (2023) (16)	Observational study	Patients with mild to moderate Alzheimer's disease (*n* = 24) and healthy controls (*n* = 15) AD: 14 male/10 female Mean age 70.3 years old Controls: 8 male/7 female Mean age 69.5 years old	Detection of subclinical epileptiform discharges	Long-term ear-EEG recordings detected epileptiform discharges in most patients with Alzheimer's disease. These discharges were not seen on short scalp EEG recordings.	Long-term outpatient ear-EEG monitoring in Alzheimer's disease.	Level 3
Musaeus et al. (2023) (17)	Observational study	Patients with dementia with Lewy bodies (*n* = 10) and healthy controls (*n* = 15) DLB: 10 male/0 female Mean age 69.9 years old Controls: 8 male/7 female Mean age 69.5 years old	Detection of subclinical epileptiform discharges	Ear-EEG recordings detected interictal epileptiform discharges in 80% of patients with dementia with Lewy bodies. Most of these epileptiform discharges occurred at night and were not seen on short scalp EEG recordings.	Long-term outpatient ear-EEG monitoring in dementia with Lewy bodies.	Level 3
Joyner et al. (2024) (14)	Validation study	Patients with refractory focal epilepsy (*n* = 20 analysed; 29 enrolled overall) 9 male/11 female Mean age 31.2 years old	Focal-onset seizure detection	The in-ear EEG device allowed clinicians to detect focal-onset seizures with high sensitivity (86.4%) compared to simultaneous video EEG. Patients also generally tolerated prolonged use well.	Clinical evaluation of prolonged ear-EEG monitoring for focal-onset seizure detection.	Level 2
Kaveh et al. (2024) (26)	Experimental study	Healthy adults (*n* = 9) 7 male/2 female Age range 18–27 years old	Drowsiness monitoring	A wireless dry EEG system showed strong results in detecting drowsiness.	Fully wireless dry-electrode ear-EEG system for drowsiness monitoring.	Level 4
Moumane et al. (2024) (27)	Experimental study	Healthy adults (*n* = 30) 10 male/20 female Mean age 26.9 years old	Signal-quality evaluation in sleep monitoring	The in-ear device demonstrated spectral patterns comparable to the 64-channel scalp cap during rest and naps, indicating its feasibility for monitoring wakefulness and sleep. However, it was more susceptible to movement-related artefacts during sleep recordings.	Comparative validation of ear-EEG against high-density scalp EEG.	Level 3
Pazuelo et al. (2024) (44)	Experimental study	Healthy adults (*n* = 18 for resting state protocol and *n* = 8 for sleep study) Resting: 10 male/8 female Mean age 28.5 years old Sleep: 7 male/1 female Mean age 27.2 years old	Signal quality evaluation	The study showed that an in-ear device could pick up clear EEG patterns during rest, eye movements, and sleep. These results correlated with those from scalp EEG recordings.	Validation of physiological signal quality across resting, wakefulness, and sleep conditions.	Level 3
Tremmel et al. (2024) (33)	Experimental study	Healthy adults (*n* = 17) 12 male/5 female Mean age 30.8 years old	Cognitive workload monitoring	A commercial in-ear EEG device demonstrated statistically significant performance in estimating cognitive workload. Although conventional EEG achieved higher accuracy, the findings support the feasibility of wearable in-ear EEG for passive brain-computer interfaces (BCIs) and routine monitoring.	Evaluation of a commercial in-ear EEG platform for cognitive workload monitoring.	Level 3
Geirnaert et al. (2025) (45)	Experimental study	Healthy adults (*n* = 15) 8 male/7 female Mean age 24.7 years old	Attention monitoring	The study found that scalp EEG is the most accurate way to assess auditory attention. However, wearable ear-EEG systems can also detect attention and could become useful, especially if used with a few extra scalp electrodes.	Ear-EEG for selective auditory attention decoding with potential hearing-aid applications.	Level 3

The table outlines the key characteristics of each study, including authors, year of publication, study design, participant characteristics, primary application, key findings, technological contribution, and level of evidence relevant to the review topic. This overview allows comparison of the included studies and provides context for the synthesis of the evidence presented in the manuscript. The quality of evidence was assessed using a review-specific framework adapted from Oxford Centre for Evidence-Based Medicine (CEBM) principles. Assessment factors included sample size, population type (healthy vs patients), if a validated comparator was used, peer-review status, and whether outcomes were functional or technical. Grade Key: Level 1 – Systematic reviews of high-quality studies. Level 2 – Well-designed clinical validation studies/strong comparative studies. Level 3 – Observational or small comparative studies. Level 4 – Case series, feasibility, experimental lab studies. Level 5 – Simulations, mechanistic studies, expert opinion.

Several limitations of this systematic review should also be acknowledged. The literature search was limited to studies indexed in PubMed and Google Scholar, and only studies published from 2018 onwards were included. Although this approach allowed the review to focus on recent developments in wearable neurotechnology, relevant earlier studies or articles indexed in other databases may have been excluded. In addition, the reviewed studies varied substantially in terms of device design, experimental protocols, and study populations, limiting the ability to directly compare findings across studies or conduct quantitative meta-analysis. Articles were also restricted to those written in English.

Future research should prioritise more rigorous evaluation of in-ear EEG technologies in both experimental and real-world environments. Head-to-head comparisons with conventional scalp EEG systems and larger prospective studies will be important for establishing reliability and clinical utility. Randomised controlled trials may also help determine whether wearable EEG monitoring improves outcomes in areas such as sleep disorders, epilepsy management, or fatigue monitoring. Future studies should also prioritise multicentre validation cohorts, standardised reporting protocols, direct comparisons between commercial and research-grade devices, and longer follow-up periods to evaluate user comfort, adherence, usability, and real-world performance. A summary of the key research priorities and their potential clinical impact is presented in Table 2.

**Table 2:** Future research priorities for the development and clinical translation of in-ear EEG.

Domain	Current challenge	Future research priority	Potential clinical impact
Signal quality	Lower signal amplitudes and reduced signal-to-noise ratio compared with scalp EEG	Improved electrode materials, adaptive filtering, and advanced signal processing	More reliable recordings in real-world settings
Electrode design	Variable electrode contact due to differences in ear anatomy	Flexible materials, custom-fit and hybrid electrode designs	Improved comfort, stability, and recording quality
Spatial coverage	Limited recording from predominantly temporal regions	Multi-electrode ear arrays and optimised electrode configurations	Improved detection of distributed brain activity
Motion artefacts	Head movement, jaw movement, and speech degrade recordings	AI-assisted artefact rejection and real-time signal correction	Greater usability during daily activities
Clinical validation	Small, heterogeneous studies with limited external validation	Large multicentre prospective validation studies	Stronger evidence supporting routine clinical use
Sleep monitoring	Limited detection of some sleep phenomena compared with scalp EEG	Direct comparisons with polysomnography and optimisation of automated sleep staging	Improved home-based sleep assessment
Epilepsy monitoring	Limited evidence for seizure localisation and seizure burden assessment	Longitudinal studies evaluating focal and generalised seizure monitoring	Improved ambulatory epilepsy management
Artificial intelligence	Limited implementation in current devices	Machine learning for automated sleep staging, seizure detection, workload assessment, and personalised monitoring	Enhanced clinical decision support and real-time interpretation
User experience	Limited evidence on comfort, adherence, and long-term wearability	Long-term human factors and implementation studies	Improved patient acceptance and sustained device use
Digital health integration	Limited integration into routine healthcare pathways	Integration with remote monitoring platforms, multimodal wearable sensors, and electronic health records	Continuous personalised neurological monitoring

The table summarises the principal challenges identified across the current literature, proposed research priorities, and their potential clinical impact. It provides a roadmap for future technological development, clinical validation, and implementation of in-ear EEG systems, highlighting key opportunities to advance wearable neurotechnology towards routine clinical and real-world applications.

Further work is also needed to improve device design and signal processing methods. Advances in flexible electrode materials, including graphene and conductive polymers such as PEDOT:PSS, may enhance electrode contact and reduce impedance. In parallel, advances in artificial intelligence and machine learning are likely to play an increasingly important role in the future of in-ear EEG. Beyond improving artefact removal and signal classification, AI may enable automated sleep staging, seizure detection, cognitive workload assessment, and personalised longitudinal monitoring, substantially enhancing the clinical utility of wearable neurotechnology. Together, these developments may support the transition of in-ear EEG from an experimental wearable technology to a practical tool for continuous brain monitoring in both clinical and everyday settings. Although important technical and clinical challenges remain, the current evidence suggests that in-ear EEG is evolving towards a practical platform for continuous neurological monitoring, with future advances in sensor technology, artificial intelligence, and clinical validation likely to determine its role within precision medicine and digital healthcare.

## Data Availability

No new or original data were generated as part of this systematic review. All data presented in this article were obtained from previously published studies and are available from the respective original sources.
